# Lung adenocarcinoma concurrent with congenital pulmonary aplasia of the right upper lobe: A case report

**DOI:** 10.3389/fonc.2022.959502

**Published:** 2022-11-02

**Authors:** Bo Min, Chu-xu Wang, Juan Liu, Li Gong, Cheng-xiang Wang, Xiao-hua Zuo

**Affiliations:** ^1^ Department of Thoracic Surgery, The Affiliated Huai’an Hospital of Xuzhou Medical University and The Second People’s Hospital of Huai’an, Huai’an, Jiangsu, China; ^2^ Department of Anesthesiology, The Affiliated Huai’an Hospital of Xuzhou Medical University and The Second People’s Hospital of Huai’an, Huai’an, Jiangsu, China; ^3^ Department of Pain Management, The Affiliated Huai’an Hospital of Xuzhou Medical University and The Second People’s Hospital of Huai’an, Huai’an, Jiangsu, China

**Keywords:** pulmonary aplasia, lung adenocarcinoma, CPD, lung abnormalities, 3D CT reconstruction

## Abstract

Lung adenocarcinoma, the most common subtype of lung cancer, has been always imposed serious threat to human health. Congenital pulmonary dysplasia (CPD) lacking typical clinical manifestations is a rare developmental anomaly. Pulmonary aplasia, the rarest subtype of CPD, may present with a variety of symptoms and is frequently associated with other abnormalities. This report describes an 81-year-old woman who presented with an irritant cough. Chest computed tomography (CT) and three-dimensional (3D) reconstruction revealed an irregular mass with a diameter of 5 cm in right lower lobe adjacent to the hilum. CT also indicated a rightward mediastinal shift and the complete absence of ipsilateral upper lobar tissue with bronchus ending in a terminal cecum, resulting in a diagnosis of pulmonary aplasia. The patient accepted lobectomy and lymph node dissection without complication, histopathologic examination combined HE staining with immunohistochemistry identified the tumor as adenocarcinoma. Three months after surgery, the patient was free of respiratory symptoms without chest pain. This report highlights the necessity of comprehensive evaluation for lung malignancy concurrent with CPD and the importance of identifying the diagnosis of pulmonary dysplasia.

## Introduction

Lung cancer is one of the most frequently diagnosed malignant tumors worldwide (1). In recent years, the incidence of lung adenocarcinoma (LUAD) has increased significantly, accounting for nearly 40% of all lung malignancies. In contrast, congenital pulmonary dysplasia (CPD), involving agenesis, aplasia, and hypoplasia, is a rare developmental anomaly of the respiratory system with unclear aetiology (2). Unilateral pulmonary dysplasia of left lung has a higher incidence and a better prognosis than dysplasia of right lung and bilateral dysplasia, especially in preterm infants. Pulmonary aplasia, defined as an undeveloped bronchial primordium combined with a complete absence of lung tissues, is the least common subtype of CPD and is frequently associated with abnormal development of the cardiovascular system, gastrointestinal tract and other organs (3–5). The present report describes an elderly woman who was diagnosed of lung adenocarcinoma in right lower lobe concurrent with pulmonary aplasia of ipsilateral upper lobe, but without other developmental abnormalities.

## Case presentation

An 81-year-old Chinese woman, non-smoker, with irritant cough for 2 months was referred to the Department of Thoracic Surgery. She initially manifested dry cough without other respiratory symptoms including fever, chest pain, wheezing and hemoptysis. There was no history of hypertension, diabetes, carcinoma, and relevant familial diseases. On physical examination, breath sounds were slightly decreased in the right lower lobe without wet or dry rales, and abnormal cardiechema was not heard. Blood examination demonstrated that complete blood count, tumor markers, blood glucose and biochemical indicator were normal. Arterial blood gas analysis showed a pH of 7.47, a PO_2_ of 74.1 mmHg, a PCO_2_ of 41.5 mmHg, an SO_2_ of 95.3% and an HCO_3_ concentration of 30.5 mmol/L on room air. Electrocardiography showed sinus rhythm with an incomplete right bundle branch block. Pulmonary function tests revealed %VC of 77.9, %FVC of 81.7, %FEV1 of 64, FEV1% of 64.42 and %DLCO of 82.9.

**Figure 1:**
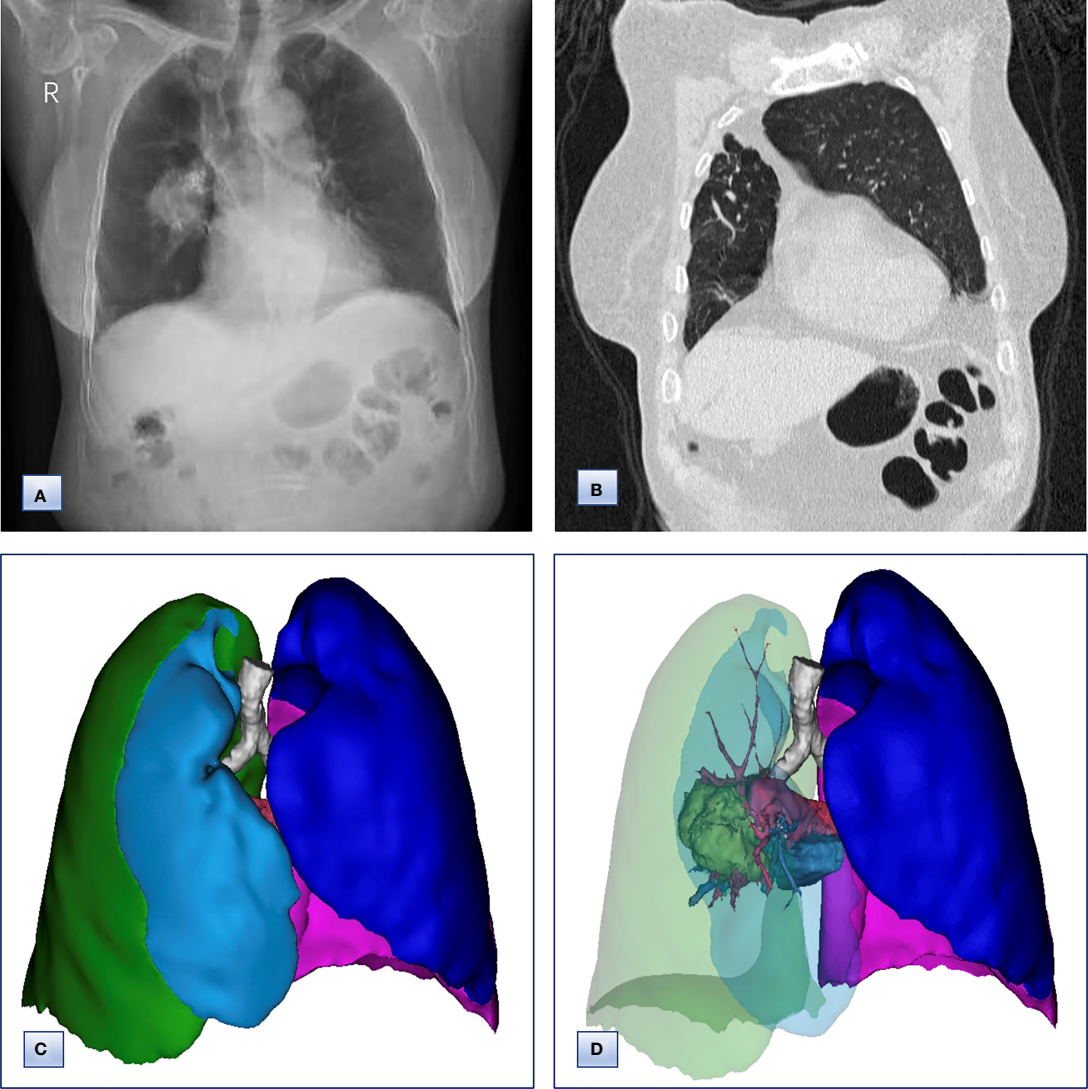
Chest radiographs, computed tomography and 3D CT reconstruction before surgery. **(A)** Chest radiographs, revealing that the trachea and mediastinum were shifted to the right. **(B)** Coronal chest CT images, indicating the absence of a horizontal fissure anterior to the aorta, with anterior herniation of the left lung crossing the midline. **(C, D)** 3D reconstruction of CT images, showing **(C)** only two lobes of the right lung and **(D)** complete absence of lung tissue from the right upper lobe, including the lung parenchyma, pulmonary vessels and bronchial tree.

**Figure 2:**
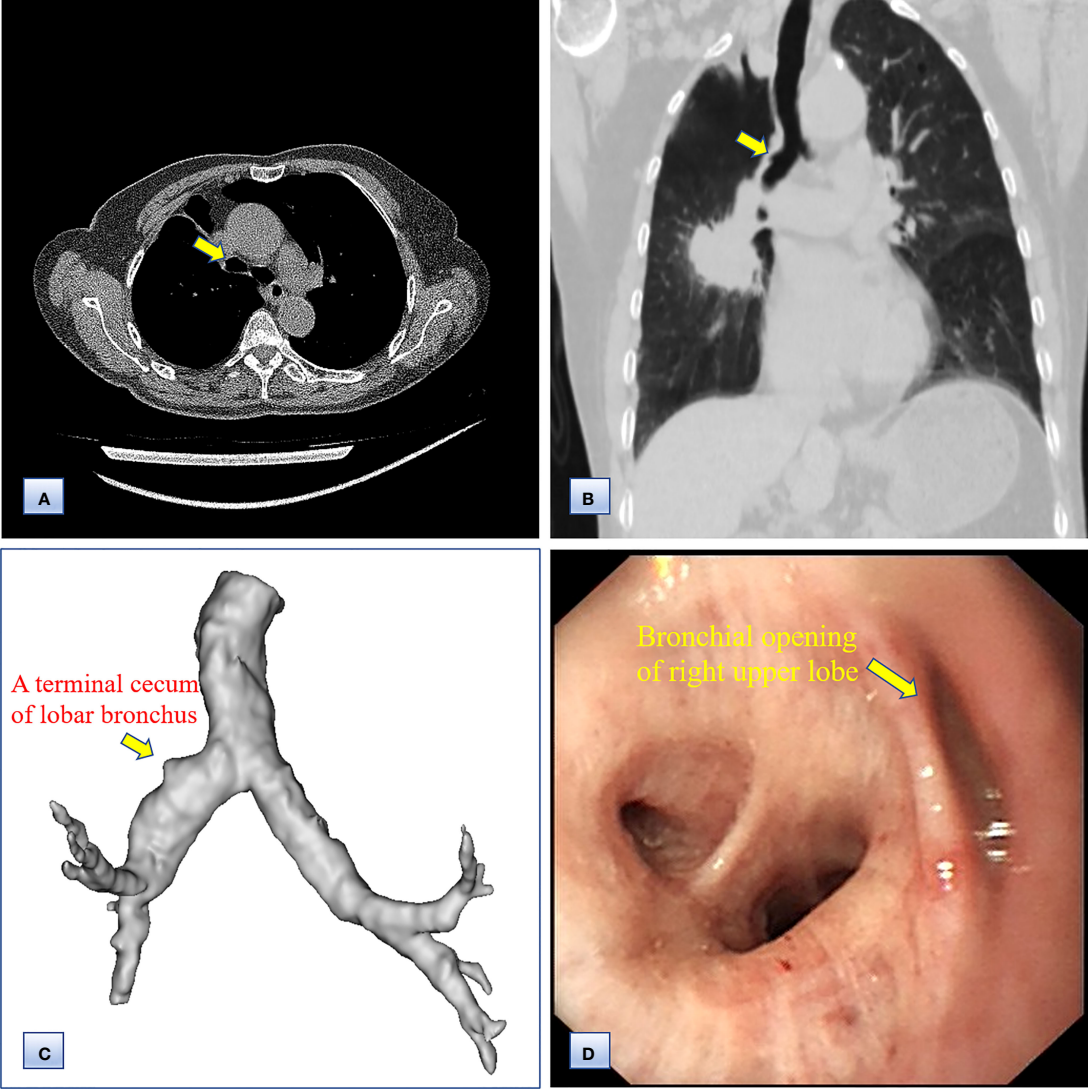
Computed tomography and bronchoscopy examination of the bronchi before surgery. **(A, B)** Axial **(A)** and coronal **(B)** chest CT images, showing the bronchus of the right upper lobe as a bud. **(C)** 3D CT reconstruction, indicating that the bronchus of the right upper lobe was a terminal cecum, and that the bronchial tree had not formed at the distal end. **(D)** Bronchoscopy examination, confirming the undeveloped bronchial primordium and ruling out a secondary obstruction.

**Figure 3:**
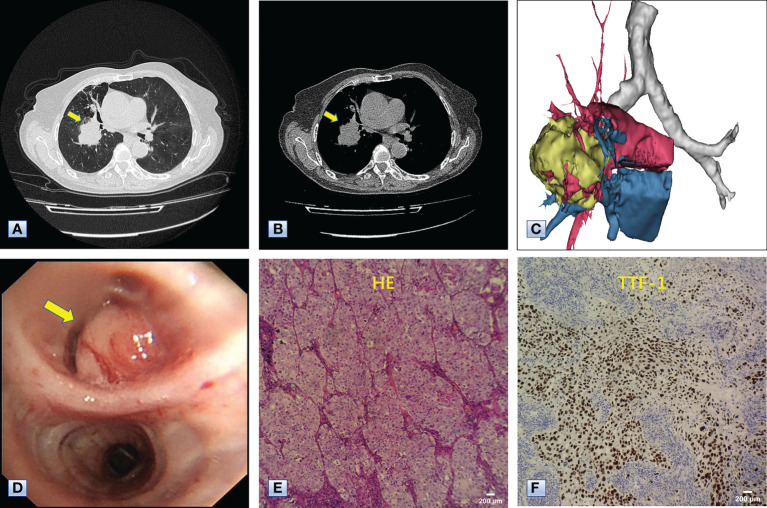
Chest CT, bronchoscopy and pathologic examination of the mass in the right lower lobe. **(A, B)** Axial images of chest CT, revealing a mass approximately 5 cm in diameter in the right lower lobe adjacent to the hilum (arrows). **(C)** 3D reconstruction revealing that the mass was adjacent to the hilum and invaded the surrounding vessels, indicating the mass was a malignancy. **(D)** Bronchoscopy results, showing a neoplasm (arrow) in the bronchus of the right basal segment. **(E)** Hematoxylin-eosin staining showing the complete absence of normal alveolar structure, replaced by growing cancer cells with enlarged nuclei and cloudy cytoplasm. **(F)** Immunohistochemical staining with antibodies to TTF-1, indicating that the tumor cells positively expressed TTF-1. The combined results of HE staining and immunohistochemistry indicated that the mass in the right lower lobe was an adenocarcinoma.

Chest computed tomography (CT) and three-dimensional (3D) reconstruction demonstrated that the trachea and mediastinum were shifted toward right, accompanied with anterior herniation of the left lung crossing the midline. There were only two lobes in the right thoracic cavity, and the entire right upper lobe, including the lung parenchyma, pulmonary vessels and bronchial tree, was completely absent (**Figure 1**). In addition, 3D CT reconstruction and bronchoscopy confirmed that the bronchus of the right upper lobe was a terminal cecum, ruling out secondary obstruction due to bronchial stenosis or intrabronchial neoplasm (**Figure 2**). The volume of each lobe calculated by Turing platform of Huiying Medical Technology before surgery were 221 ml of right middle lobe, 1232 ml of right lower lobe, 834 ml of left upper lobe and 642 ml of left lower lobe. CT images also revealed an irregular mass of hyperdense with a diameter of 5 cm in right lower lobe adjacent to the hilum, surrounding the bronchus and arteries of lower lobe, as well as enlargement of subcarinal and lobar nodes. Bronchoscopy examination discovered a neoplasm in the right basal section, and the pathological type of biopsy was adenocarcinoma (**Figures 3A-D**). Bone scintigraphy, brain MRI and abdominal CT excluded tumor metastases. Echocardiography indicated mild regurgitation of mitral valves and a slight increase of pulmonary tension which were caused by aging of organs, with the normal location and structure of heart and great vessels, suggesting that the pulmonary aplasia of this patient was not combined with cardiovascular malformations.

**Figure 4:**
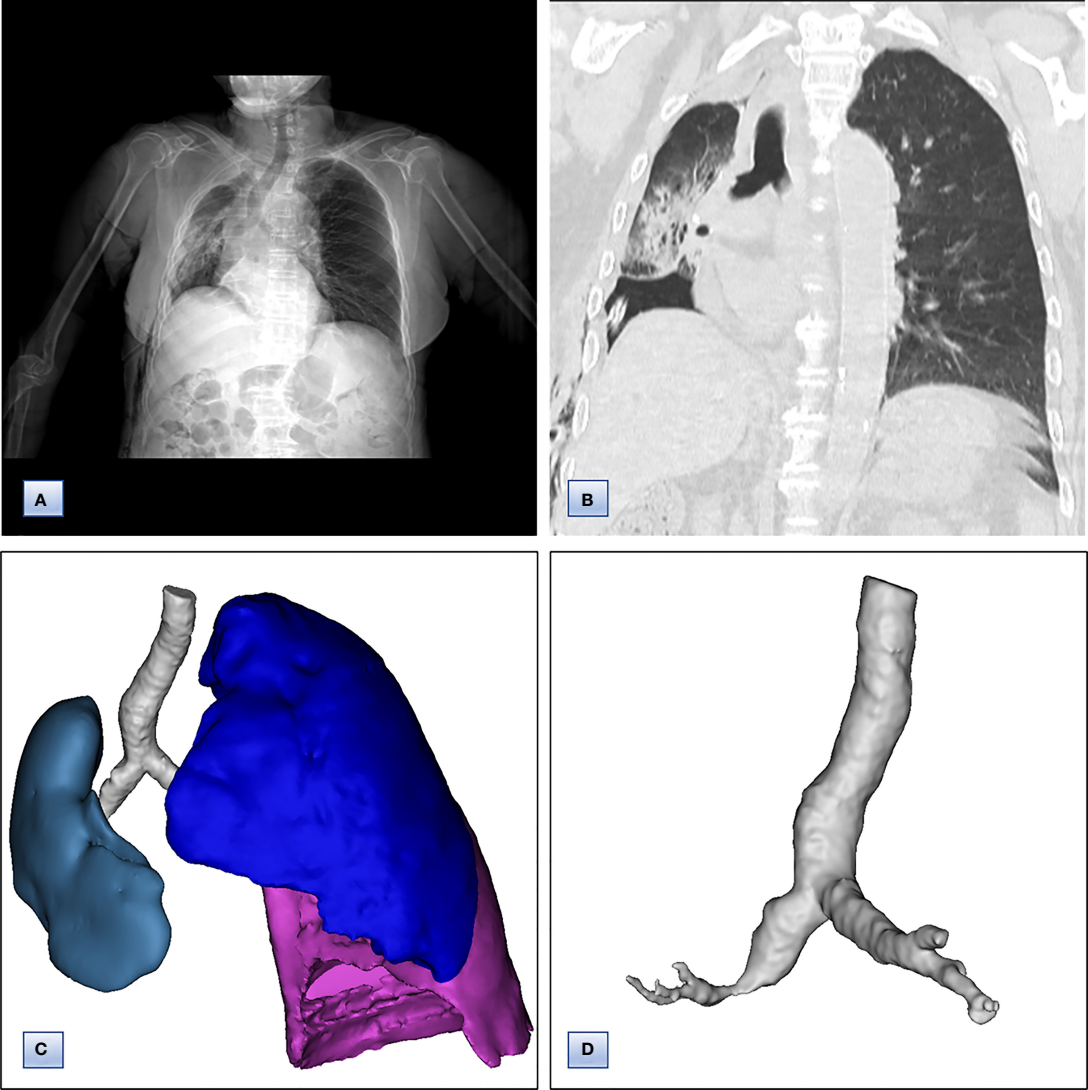
Chest computed tomography and7 3D CT reconstruction after surgery. **(A)** Chest CT, revealing that the trachea and mediastinum were more obviously shifted to right than that before surgery. **(B)** Coronal chest CT images, indicating adequate recruitment of the right middle lobe. **(C, D)** 3D reconstruction of CT images, showing **(C)** only middle lobe in right thoracic cavity and **(D)** unobstructed bronchus of right middle lobe.

The Multidisciplinary team including thoracic surgery, anesthesiology, respirology, oncology and radiology, reached a consensus on the treatment of neoadjuvant chemotherapy or operation. The patient refused adjuvant treatment before operation and requested radical surgery of lung cancer. She underwent lobectomy of right lower lobe and lymph node dissection under general anesthesia. A tumor measured 4.5×4×3.5 cm in size was located near the bronchial stump of right lower lobe. The cut surface showed a solid tumor with greyish white color. Immunohistochemical staining of tumor indicated that NapsinA, TTF-1, CK7 were positive expression and CgA, Syn, CD56 were negative expression (**Figures 3E, F**). Lymph node involvement was as follows: Group 2 lymph nodes (0/1), Group 4 lymph nodes(0/1), Group 7 lymph nodes (2/3), “Group 9 lymph nodes” (0/1), “Group 10 lymph nodes” (2/3), “Group 11 lymph nodes” (1/1) and “Group 12 lymph nodes” (2/2). The combination of HE and immunohistochemical studies lead to a diagnosis of right lung adenocarcinoma (pT2N2M0), which was consistent with preoperative pathological type and staging. Postoperative chest CT and 3D reconstruction indicated more pronounced mediastinal shift toward right and adequate recruitment of the right middle lobe (**Figure 4**). The volume of each lobe by Turing platform after surgery were 406 ml of right middle lobe, 888 ml of left upper lobe and 762 ml of left lower lobe. The volume of each lobe after operation was significantly increased compared with that before operation. The patient felt well after surgery without complications and refused genetic testing of tumor, targeted therapy, chemotherapy. At follow-up 3 months after discharge by telephone, the patient reported no chest pain or irritant cough.

## Discussion

To our knowledge, this is the first report of lung cancer concurrent with congenital pulmonary aplasia. Lung cancer is the most common type of malignancy and the leading cause of cancer-related deaths around the world, with more than 2 million new diagnosed and 1.6 million died per year (6). About 85% of lung cancer is non-small cell lung cancer (NSCLC), and the incidence of lung adenocarcinoma (LUAD) is increasing year by year (7). Despite the availability of more individualized treatments, including surgery, chemoradiotherapy, and molecular targeted drugs, the prognosis of NSCLC remained poor, with 5-year overall survival rate at 17.7% (8).

In contrast, pulmonary developmental arrest is an exceedingly uncommon congenital defect, with a prevalence of approximately 1–2 per 200,000 births (9). Congenital pulmonary dysplasia has been classified into three subtypes: agenesis (the complete absence of lung tissue), aplasia (a main bronchus ending in a terminal cecum) and hypoplasia (a bronchus with rudimentary pulmonary tissue). Because of their similar radiography appearance, pulmonary agenesis, aplasia, and hypoplasia have been termed “lung agenesis-hypoplasia complex”, which can be present in an entire lung, a lobe or a segment (10).

About 10-20% of newly diagnosed lung cancer patients had a clinical stage of IIIA/N2 before operation. Although the therapeutic strategies for stage IIIA/N2 NSCLC patients are controversial, numerous research have verified that surgery provided longer survival compared with chemotherapy or chemoradiotherapy, especially for patients with lymph nodes smaller than 3 cm (11). Common postoperative complications of lobectomy included pulmonary embolus, respiratory failure, bronchopleural fistula, hemothorax, pneumothorax and pneumonia. The patient underwent radical surgery for lung cancer without any postoperative complications, and refused chemotherapy and targeted therapy after surgery.

Pulmonary aplasia, characterized by a rudimentary bronchus without lung tissue, is the least common subtype. However, pulmonary aplasia and agenesis, which are considered as an entity, are usually congenital and resulting in a similar anomaly due to the complete absence of lung (12). The pathogenesis of pulmonary dysplasia is still unclear. Its onset, between the fourth and fifth week of gestation, may be caused by a duplication of the distal upper arm of chromosome 2, de-regulating calcium signal-related proteins and mitochondrial bioenergetic dysfunction (13–16). Patients with pulmonary aplasia were frequently unilateral, with pulmonary dysplasia of left lung being of higher incidence and lower mortality.

Most patients with congenital pulmonary aplasia were diagnosed in infancy or childhood, with few remaining asymptomatic into adulthood. About 50% of patients with unilateral lung dysplasia died within 5 years of birth, whereas bilateral pulmonary dysplasia was life-threatening. Furthermore, neonates with irreversible pulmonary underdevelopment may require long-term cardiopulmonary support (17). The clinical manifestations of pulmonary aplasia including cough, exercise intolerance, recurrent respiratory infections, wheezing and dyspnea were various depending on the number of alveoli. Bronchitis was the most frequent symptom in patients with pulmonary aplasia for the bronchial cecum serving as a source of infections (18). In addition, few patients remaining asymptomatic until adulthood were incidentally diagnosed by imaging examination, possibly due to the developmental defect of less lung tissue without cardiovascular abnormalities.

The diagnosis of pulmonary aplasia which is extremely challenging due to the lack of specific symptoms and signs requires a suspicious medical history, careful physical examination, and comprehensive auxiliary examinations. Physical examination may detect an asymmetrical chest with tracheal deviation and abnormal breath sounds (19). Chest radiographs, CT scan, magnetic resonance imaging (MRI), bronchoscopy and bronchography are critical diagnostic approaches, with 3D CT reconstruction being the most important. Chest radiographs of pulmonary aplasia usually revealed diminished radiolucency and the shift of mediastinum, while CT images can distinguish the absence of lung tissue from atelectasis (20, 21). CT examination of the patient indicated a mediastinal shift toward right, the complete absence of right upper lobe and anterior herniation of left lung, coexisting with an irregular mass in right lower lobe adjacent to the hilus. Moreover, 3D CT reconstruction and bronchoscopy ascertained the bronchus of the right upper lobe as a blind pouch, a indicative of congenital undeveloped bronchial primordium, and ruled out secondary atelectasis caused by tumors, tuberculosis, infection and swollen lymph nodes.

Because patients with unilateral pulmonary aplasia have a significant amount of normal lung tissue, the occurrence and severity of associated malformations may become the most important factor affecting prognosis. Multiple developmental abnormalities have been reported in patients with pulmonary dysplasia, including coarctation of the aorta, esophageal atresia, pulmonary artery atresia, lung herniation, ventricular septal defect and tetralogy of Fallot (3, 21–23). Ultrasonography excluded the abnormalities of heart, aorta, main pulmonary artery and abdominal organs. Current consensus is that prophylactic surgery is not required in asymptomatic patients with pulmonary dysplasia. The patient underwent a lobectomy due to the tumor in right lower lobe instead of pulmonary dysplasia in right upper lobe. Pathological examination of HE staining and immunohistochemistry revealed that the tumor was adenocarcinoma with regional lymph node metastasis. Activating mutations in epidermal growth factor receptor (EGFR) have been found in approximately 50-60% of Asian female patient, and this non-smoking female patient refused genetic testing of the tumor. Eight previous reports about patients with right pulmonary aplasia have been published (**Table 1**), whereas, to our knowledge, pulmonary aplasia of one lobe concurrent with ipsilateral lung cancer has not been reported previously.

**Table 1:** Summary of right pulmonary aplasia of the scalp.

No.	Authors, year	Age, Gender	Onset age	Location	Symptom	Surgery	Combined with lung cancer	Other abnormalities	Outcome
1	D Ryland et al. (1)	3m, N/A	1m	Right whole lung	Tachypnoea	No	No	The presence of 13 ribs	Death
2	T B Buxi et al. (2)	4d, M	24 hours after birth	Right whole lung	Respiratory distress	Yes	No	None	Death
3	Thomas Nowotny et al. (3)	4m, M	2m	Right whole lung	An airway infection	Yes	No	None	Clinically stable
4	Peter Lee et al. (4)	10m, M	From birth	Right whole lung	Respiratory distress	Yes	No	Patent ductus arteriosus and bronchopulmonary sequestration	Favourable
5	Se Hwan Kwon et al. (5)	N/A, F	Incidental	Right whole lung	Asymptomatic	No	No	None	Favourable
6	Arshad et al. (6)	20y, F	17y	Right whole lung	Dyspnea on exertion	No	No	MRKH syndrome	Favourable
7	Augusto et al. (7)	5y, F	Incidental	Right whole lung	Asymptomatic	Yes	No	Right diaphragmatic hernia	Favourable
8	Pandey NN et al. (8)	7m, N/A	From birth	Right whole lung	Respiratory distress and feeding difficulties	No	No	Diaphragmatic hernia and tetralogy of Fallot	Favourable
9	Present case						Yes		

MRKH, Mayer-Rokitansky- Küster-Hauser; M, male; F, female; m, months; N/A, not available.

In summary, this report described an elderly woman with adenocarcinoma in right lower lobe concurrent with pulmonary aplasia of ipsilateral upper lobe, and emphasized the characteristic changes of CT images and bronchoscopy. Asymptomatic patients with pulmonary aplasia of one lobe do not require surgical treatment, and can tolerate lobectomy, which plays a vital role in the comprehensive evaluation of lung cancer concurrent with pulmonary dysplasia.

## Data availability statement

The raw data supporting the conclusions of this article will be made available by the authors, without undue reservation.

## Ethics statement

The studies involving human participants were reviewed and approved by the Ethics Committee of the Affiliated Huai’an Hospital of Xuzhou Medical University. The patients/participants provided their written informed consent to participate in this study. Written informed consent was obtained from the individual(s) for the publication of any potentially identifiable images or data included in this article.

## Author contributions

All authors made a significant contribution to this report. BM and C-XuW drafted the manuscript. X-HZ revised the manuscript. All authors contributed to the article and approved the submitted version.

## Funding

This study was funded by the Huai'an Health Project (No.HAWJ202115).

## Acknowledgments

The authors thank the patient and her family.

## Conflict of interest

The authors declare that the research was conducted in the absence of any commercial or financial relationships that could be construed as a potential conflict of interest.

## Publisher’s note

All claims expressed in this article are solely those of the authors and do not necessarily represent those of their affiliated organizations, or those of the publisher, the editors and the reviewers. Any product that may be evaluated in this article, or claim that may be made by its manufacturer, is not guaranteed or endorsed by the publisher.
